# Identification of glomerular and podocyte-specific genes and pathways activated by sera of patients with focal segmental glomerulosclerosis

**DOI:** 10.1371/journal.pone.0222948

**Published:** 2019-10-03

**Authors:** Lilian Otalora, Efren Chavez, Daniel Watford, Lissett Tueros, Mayrin Correa, Viji Nair, Philip Ruiz, Patricia Wahl, Sean Eddy, Sebastian Martini, Matthias Kretzler, George W. Burke, Alessia Fornoni, Sandra Merscher

**Affiliations:** 1 Katz Family Division of Nephrology and Hypertension, Department of Medicine, University of Miami Miller School of Medicine, Miami, Florida, United States of America; 2 Peggy and Harold Katz Family Drug Discovery Center, University of Miami Miller School of Medicine, Miami, Florida, United States of America; 3 Department of Internal Medicine, University of Miami/Jackson Memorial Hospital, Miami, Florida, United States of America; 4 Department of Surgery, University of Miami Miller School of Medicine, Miami, Florida, United States of America; 5 Miami Transplant Institute, University of Miami Miller School of Medicine, Miami, Florida, United States of America; 6 Department of Pathology, University of Miami Miller School of Medicine, Miami, Florida, United States of America; 7 Division of Nephrology, Department of Internal Medicine, University of Michigan School of Medicine, Ann Arbor, Michigan, United States of America; 8 Diabetes Research Institute, University of Miami Miller School of Medicine, Miami, Florida, United States of America; University of Houston, UNITED STATES

## Abstract

Focal segmental glomerulosclerosis (FSGS) accounts for about 40% of all nephrotic syndrome cases in adults. The presence of several potential circulating factors has been suggested in patients with primary FSGS and particularly in patients with recurrent disease after transplant. Irrespectively of the nature of the circulating factors, this study was aimed at identifying early glomerular/podocyte-specific pathways that are activated by the sera of patients affected by FSGS. Kidney biopsies were obtained from patients undergoing kidney transplantation due to primary FSGS. Donor kidneys were biopsied pre-reperfusion (PreR) and a subset 1–2 hours after reperfusion of the kidney (PostR). Thirty-one post reperfusion (PostR) and 36 PreR biopsy samples were analyzed by microarray and gene enrichment KEGG pathway analysis. Data were compared to those obtained from patients with incident primary FSGS enrolled in other cohorts as well as with another cohort to correct for pathways activated by ischemia reperfusion. Using an ex-vivo cell-based assay in which human podocytes were cultured in the presence of sera from patients with recurrent and non recurrent FSGS, the molecular signature of podocytes exposed to sera from patients with REC was compared to the one established from patients with NON REC. We demonstrate that inflammatory pathways, including the TNF pathway, are primarily activated immediately after exposure to the sera of patients with primary FSGS, while phagocytotic pathways are activated when proteinuria becomes clinically evident. The TNF pathway activation by one or more circulating factors present in the sera of patients with FSGS supports prior experimental findings from our group demonstrating a causative role of local TNF in podocyte injury in FSGS. Correlation analysis with clinical and histological parameters of disease was performed and further supported a possible role for TNF pathway activation in FSGS. Additionally, we identified a unique set of genes that is specifically activated in podocytes when cultured in the presence of serum of patients with REC FSGS. This clinical translational study supports our prior experimental findings describing a potential role of the TNF pathway in the pathogenesis of FSGS. Validation of these findings in larger cohorts may lay the ground for the implementation of integrated system biology approaches to risk stratify patients affected by FSGS and to identify novel pathways relevant to podocyte injury.

## Introduction

Focal segmental glomerulosclerosis (FSGS) is a heterogeneous disease accounting for about 40% of all nephrotic syndrome cases in adults[1, 2] and is thought to be the result of podocyte injury. The renal lesions found in FSGS can be the result of an unknown cause (idiopathic or primary FSGS) or they can occur as a result of genetic defects (genetic FSGS), previous glomerular injury, viral infections, drugs or toxins (secondary FSGS)[3]. The diagnosis of FSGS is usually made upon renal biopsy with confirmation of the presence of sclerosis in parts of some glomeruli by light microscopy as well as, in most cases of primary FSGS, diffuse foot process effacement by electron microscopy [4].

In half of the patients affected by primary FSGS, steroid treatment leads to remission while in the other half, steroid resistance results in kidney failure after 5 years. Although kidney transplantation is the favored form of renal replacement therapy, recurrence rates ranging from 6–65% were reported[5–9], underscoring the need to develop new therapeutic strategies for the treatment of patients with primary FSGS.

Research suggests that the pathogenesis of primary FSGS is due to a circulating permeability factor(s) present in the sera of patients, yet the actual factor remains to be identified and its existence remains elusive [10]. A seminal study by V. Savin demonstrated that serum from patients with FSGS increases glomerular permeability to albumin [11]. While the permeability activity was highest when serum from patients with post-transplant recurrence (REC) was used, plasma from patients with non-recurrent FSGS (NON REC) increased albumin permeability as well but with a lower level of activity. Similarly, not all sera from patients with REC increased permeability at high levels. The authors concluded that a factor present in the serum from patients with FSGS is strongly associated with REC after renal transplantation[11]. Among factors that may increase glomerular permeability to albumin are vascular permeability factor (VPF), hemopexin, soluble urokinase receptor (suPAR), cardiotrophin-like cytokine-1 (CLC-1), other cytokines[12], and more recently CASK[13]. In other studies, it was demonstrated that exposure of mouse podocytes to a combination of two circulating factors, i.e. suPAR and TNF, is sufficient to mimic some of the effects caused by exposure to serum or plasma from patients with REC of FSGS[14, 15], including an increased steady-state abundance of TRPC6 at the cell surface, the activation of alpha v beta 3 integrin signaling and decreased podocin expression. Interestingly, an effect on podocin expression was only seen in suPAR but not in TNF treated mouse podocytes, while TNF treatment increased the effects of suPAR on podocin expression and TRPC6 localization, suggesting that multiple circulating factors might contribute to primary FSGS.

Irrespectively of the nature of specific permeability factors, several clinical and experimental data suggest an implication of the TNF pathway in the pathogenesis of podocyte injury in FSGS. Elevated levels of serum TNF have been described in patients with nephrotic syndrome[16] and a beneficial role of TNF inhibition was observed in a subset of pediatric patients with FSGS[17, 18]. Consistent with these data, we recently established a causative role of renal TNF in lipid-dependent podocyte injury in experimental FSGS[19]. The observation that glomerular but not serum TNF levels correlated with loss of eGFR in this study suggested that, regardless of the nature of one or more circulating factors present in the serum of patients with FSGS, the renal intrinsic activation of the TNF pathway contributes to disease pathogenesis and/or progression in FSGS[19], or that multiple circulating factors contribute to the pathogenesis of primary FSGS and thus correlations between a single circulating factor and a clinical phenotype might not always be evident[20]. In support of this hypothesis, Mariani et al. (2018) reported that patients at high risk for FSGS progression (cluster 3 patients) are characterized by a transcriptional profile consistent with TNF activation that correlates with eGFR loss [21]. Finally, Chung et al. (2019) demonstrated that activation of the TNF pathway occurs in podocytes of patients with FSGS[22]. While TNF seems to play a role, a more unbiased approach is needed to allow for the identification of other pathways that are activated in normal kidneys exposed to the sera of patients affected by FSGS as well as in kidney biopsies of patients with clinically evident FSGS.

Gene expression studies using kidney biopsies from patients with FSGS have been performed in order to identify gene expression signatures in patients with FSGS that can be correlated with clinical phenotypes or therapeutic response[23–26]. However, the experimental design of these studies did not allow for the specific identification of pathways activated by the sera of affected patients.

In this study, we used a unique approach that allows for the identification of pathways that are activated by circulating factors present in the sera of kidney transplant recipients with FSGS immediately after kidney transplantation, i.e. we performed microarray analysis of glomerular mRNA isolated from Pre-and PostR biopsies of patients transplanted for biopsy-proven primary FSGS. While PreR biopsies were obtained from the donor kidney before transplantation, the PostR biopsy was obtained 1–2 hours after transplantation and reperfusion of the kidney, thus allowing for the identification of pathways activated early after the exposure of the kidney to the sera of patients with primary FSGS. As a limited number of PostR biopsies were available from patients that developed REC after transplant, we used sera from a larger number of patients with REC FSGS that we had stored and performed transcriptome analysis of normal human podocytes that were contacted with the serum from patients with or without REC of FSGS with the goal to identify pathways that may differentiate REC from NON REC disease.

## Material and methods

### Patient enrollment

Thirty-nine patients with biopsy proven primary FSGS were enrolled under an Institutional Review Board (IRB) of the University of Miami-approved study protocol (#20100498) at the University of Miami, ClinicalTrials.gov, NCT01164098 over a four-year period and followed up for 12 months. Written consent was obtained from all patients and patients were offered the opportunity to participate in the study after a full explanation of the study by allowing the utilization of protocol kidney biopsies, serum and urine samples for the experimental studies. All research involving human participants was conducted according to the principles expressed in the Declaration of Helsinki.

### Patient history and characteristics at baseline and during follow-up

All patients were originally diagnosed with nephrotic syndrome (NS). Fifty-six percent (22/39) of the patients were treated with corticosteroids after NS diagnosis, 18% (7/39) did not receive corticosteroid treatment and for 26% (10/39) no information about the initial treatment was available. Of the patients that were treated with corticosteroids after the initial NS diagnosis, 63% (14/22) did not respond to the treatment (steroid-resistant NS, SRNS) while 32% (7/22) responded initially but ultimately had REC of FSGS and/or progressed to ESRD (steroid-sensitive NS, SSNS with frequent relapse). For one patient no information with regard to the treatment response was available. Exclusion criteria and sample procurement details are indicated in S1 File. Four out of 39 patients developed nephrotic range proteinuria (REC) in the follow-up period. Several others developed a variable degree of proteinuria. The graft survival was 100% at the end of the study period. In addition to these well characterized 39 patients, 10 high risk patients with FSGS that were enrolled in a prior study[5] were also included in this study, and available sera and/or kidney biopsies were utilized for molecular profiling studies. Among these 10 patients, 6 developed REC FSGS.

### Kidney biopsy procurement and processing

Donor kidneys were biopsied pre-reperfusion (PreR) and a subset 1–2 hours PostR. Wedge biopsies of donor kidneys were obtained intra-operatively during kidney transplantation (S1A Fig). Biopsy sites were oversewn. Each biopsy sample was immediately cut in four pieces. Pieces were processed as illustrated in S1B Fig. One piece was placed in 2.5% glutaraldehyde fixative for Electron Microscopy (EM) analysis. A second piece was placed in 10% buffered formalin solution for histological analysis. A third piece was submerged in Michel’s transport medium (55 g NH_4_SO_4_ per 100 ml 0.9% NaCl, pH 7.4) followed by embedding in optimum cutting temperature (*O*.*C*.*T*.) formulation (Tissue-Tek) for immunofluorescence. Finally, the last piece placed in saline solution, then transferred to RNA later (Qiagen) for subsequent mRNA extraction and microarray analysis (S1 Fig).

### Other cohorts analyzed in this study

Other microarray data implemented in this study were obtained from the Nephrotic Syndrome Study Network (NEPTUNE) cohort, the European Renal Biopsy cDNA Bank (ERCB) and the “Alberta” study[27]. Data for the ERCB and Alberta studies are publicly available at https://www.ncbi.nlm.nih.gov/geo/query/acc.cgi?acc=GSE104948 and https://www.ncbi.nlm.nih.gov/geo/query/acc.cgi?acc=GSE37838, respectively. Partial datasets from the NEPTUNE study with relevance to this manuscript are provided in the S1 Table of the manuscript. A more detailed description of these cohorts is available in the S1 File of this manuscript.

### Microarray analysis (MA) of kidney biopsies and of human podocytes contacted with serum obtained from patients with FSGS

Kidney biopsy tissue for microarray analysis was manually microdissected into glomerular and tubulointerstitial compartments. RNA was extracted using the AllPrep RNA/DNA Micro 80284 Kit (Qiagen) following manufacturer’s instructions. cDNA was prepared from 10-15ng total RNA (NuGen RNA cDNA Pico SL WTA system V2). 2.5μg of cDNA was biotinylated (NuGEN Encore Biotin Module, P/N M01111 v6) and profiled on Affymetrix Human Gene ST 2.1 array plates (Affymetrix).

A total of 36 PreR and 31 PostR biopsy samples was available from a total of 49 patients; 39 patients were enrolled under this study protocol and 10 additional high-risk patients that participated in a previous study[5]. The 36 PreR biopsy samples included 6 patients that developed REC while 30 patients were NON REC. Of the 31 PostR biopsies, 5 samples were obtained from patients that developed REC while 26 patients were NON REC. For microarray analysis, samples were separated into samples from patients with REC and NON REC. cDNAs were profiled on Affymetrix Human Gene ST 2.1 array plates (Affymetrix, Santa Clara, CA)(n = 1). ERCB samples were profiled on the Affymetrix Human Genome U133 A and Plus 2.0 arrays. The Alberta dataset was profiled on the Affymetrix Human Genome U133 Plus 2.0 Array[27].

Affymetrix*.cel image files were obtained using the Affymetrix GeneChip software and sample/chip quality was assessed based on multiple quality metrics as implemented in the R statistical platform. RMA (Robust Multi-Array Average) (Irizarry et al., 2003) normalization of CEL files was performed in R using custom CDF version 19 and modified Affymetrix_1.44.1 package from BrainArray (http://brainarray.mbni.med.umich.edu/brainarray/default.asp). The normalized expression matrix was log2 transformed for all downstream analyses.

Significance Analysis of Microarray (SAM) as implemented in MeV from the TiGR suite (http://www.tm4.org/mev.html) method was used to perform differential analysis. The significance threshold was set at FDR <= 0.10. To identify podocyte-specific markers contributing to the REC of FSGS, microarray analysis of normal human podocytes contacted with the sera from six patients with REC and from four patients without recurrence (NON REC) was performed (n = 1). The sera used as well as the method to culture human podocytes in the presence of patients sera were previously described in detail[5]. Analysis of gene expression results was performed on Illumina. The calculated signals and detection calls were imported into Agilent’s GeneSpring GX 10 for processing, using the HumanWG-6_V3_0_R1_11282955_A chip technology. Normalization, Quality control, Statistical analysis, and Fold change analysis were performed in GeneSpring. The resulting gene list was then further analyzed using GeneSet Analyzer for the purposes of Pathway (KEGG) and Gene Ontology (GO) analyses. The 33,346 entities were analyzed to determine the statistically significant differentially expressed genes. Analysis was done using an unpaired T-Test and multiple correction testing. A p-value of <= 0.05 was considered significant. To further reduce the rate of false positives, multiple correction testing (Benjamini-Hochberg) was employed.

### Identification of commonly dysregulated genes from different microarray studies

To identify genes commonly dysregulated between two or more different microarray studies, differentially regulated genes obtained within each analysis were compared using the Gene List Venn Diagram software available at http://genevenn.sourceforge.net.

### Gene enrichment and KEGG pathway analysis

To identify genes enriched in a selected list of genes, gene enrichment analysis followed by KEGG pathway analysis was performed using the ToppGene suite (https://toppgene.cchmc.org/enrichment.jsp). Selected pathways from the top 20 pathways identified at p<0.05 are shown, pathways are listed in order of significance.

### Statistical analysis and correlation analysis

Prism 6 for Mac OS X (GraphPad Software, Inc.) was used for statistical analysis. Normalized and log2 transformed microarray data were used to test for correlation between biomarkers and clinical outcomes of interest or glomerular TNF gene expression. The Pearson correlation coefficients were determined for a 95% confidence interval; results were considered statistically significant when p<0.05.

## Results

### Patient demographics/characteristics and clinical parameters

Thirty-nine patients with biopsy-proven primary FSGS were enrolled and followed for 12 months (NCT0116498). All patients were initially diagnosed with nephrotic syndrome and did not self-report any family history of renal disease. Baseline demographics are listed in Table 1, clinical parameters collected at baseline and after transplantation are shown in Table 2. Only 4/39 patients had REC, all other patients had no proteinuria or subnephrotic proteinuria (NON REC). Out of the four patients with REC, two were teenagers and had immediate REC while the two other patients were adults and REC occurred 3 months post-transplantation. Additional ten patients with high-risk of REC of FSGS from a previous study were also included[5]. Among these 10 patients, 6 developed REC of FSGS.

**Table 1:** Baseline demographics of the patients.

Parameter	Mean ± SD (N = 39)	Mean ± SD (N = 6)	Mean ± SD (N = 45)
Age (years)	35.10 (± 13.84)	24.71(± 9.44)	33.52 (± 13.68)
Weight (kg)	72.27 (±19.22)	63.26 (±21.32)	70.77 (±19.60)
Race (W/B/O)	27/11/1 (69%/28%/3%)	5/2 (71.4%/28.6%)	32/13/1 (69.5%/28.3%/2.2%)
Ethnicity (Hispanic/Non-Hispanic)	16/23 (41%/59%)	2/5 (28.6%/71.4%)	18/28 (39.1%/60.9%)
Gender (M/F)	26/13 (67%/33%)	3/4 (42.8%/57.2%)	29/17(63%/37%)
Donor (LD/DD)	25/14 (64%/36%)	7/0 (100%/0%)	32/14 (70%/30%)
Time to ESRD	4.25 (±4.18)	4.82 (±3.54) (N = 5)	4.44 (±4.33)

N = 45 with N = 39 enrolled in this study, N = 6 enrolled in a previous study[5]. End-stage renal disease (ESRD) is defined as GFR < 15 ml/min/1.73 m^2^ or dialysis.

**Table 2:** Clinical parameters of the patients.

Parameter	Baseline (Mean ± SD)	1 months (Mean ± SD)	3 months (Mean ± SD)	12 months (Mean ± SD)
Serum albumin (g/dL)	3.74 ± 0.65 (3.96 ± 0.70)	4.058 ± 0.56^1^	4.14 ± 0.49	4.16± 0.33^2^
Serum creatinine (mg/dL)	8.09 ± 3.54 (9.76 ± 6.33)	1.48± 0.95	1.28 ± 0.36	1.36 ± 0.50
BUN (mg/dL)	49.62 ± 19.26 (38.57 ± 14.55)	24.73 ± 10.30^3^	21.16 ± 6.61	23.02 ± 8.55
GFR (ml/min/1.73m^2^)	9.13 ± 4.85 (10.50 ± 8.58)	60.13 ± 21.26	68.15 ± 31.29	62.66 ± 27.34^3^
Protein/creatinine ratio UPCR (g/g)	10.77 ± 41.44 N/A	1.38 ± 4.72	2.48 ± 8.73^4^	0.41 ± 0.61^5^
Total Cholesterol (mg/dL)	159.18 ± 38.98^6^ (139.60 ± 70.54)	N/A	172.29 ± 58.74^7^	176.29 ± 42.33^8^
HDL (mg/dL)	45.91 ± 12.9^9^ (29.20 ± 15.16)	N/A	46.53 ± 8.51^10^	45.19 ± 14.21^11^
LDL (mg/dL)	76.61 ± 23.11^12^ (68.80 ± 47.11)	N/A	87.76 ± 43.51^13^	96.26 ± 30.13^14^
Steroids (Y/N/U)	22/7/10 (56%/18%/26%) 5/1 (83%/17%)			
Diabetes Medication (Y/N)	3/36 (8%/92%) 0/6 (0%/100%)	3/36 (8%/92%)	3/35 (8%/92%)	2/32 (6%/94%)
Lipid medication (Y/N)	9/30 (23%/77%) 1/5 (17%/83%)	9/30 (23%/77%)	8/30 (21%/79%)	8/26 (24%/76%)
Antihypertensive medication (Y/N)	35/4 (90%/10%) 6/0 (100%/0%)	35/4 (90%/10%)	32/6 (84%/16%)	28/6 (82%/18%)
Graft survival at 1 year	100%	100%	100%	100%

N = 45 for clinical parameters at baseline, with N = 39 enrolled in this study, N = 6 enrolled in a previous study[5]. N = 39 for clinical parameters at 1, 3 and 12 months.

^1^N = 38,

^2^N = 38,

^3^N = 38,

^4^N = 37,

^5^N = 38,

^6^N = 34,

^7^N = 17,

^8^N = 31,

^9^N = 34,

^10^N = 17,

^11^N = 31,

^12^N = 34,

^13^N = 17,

^14^N = 31.

Y = yes, N = no, U = unknown.

### Cohorts and strategy of analysis

**Fig 1:**
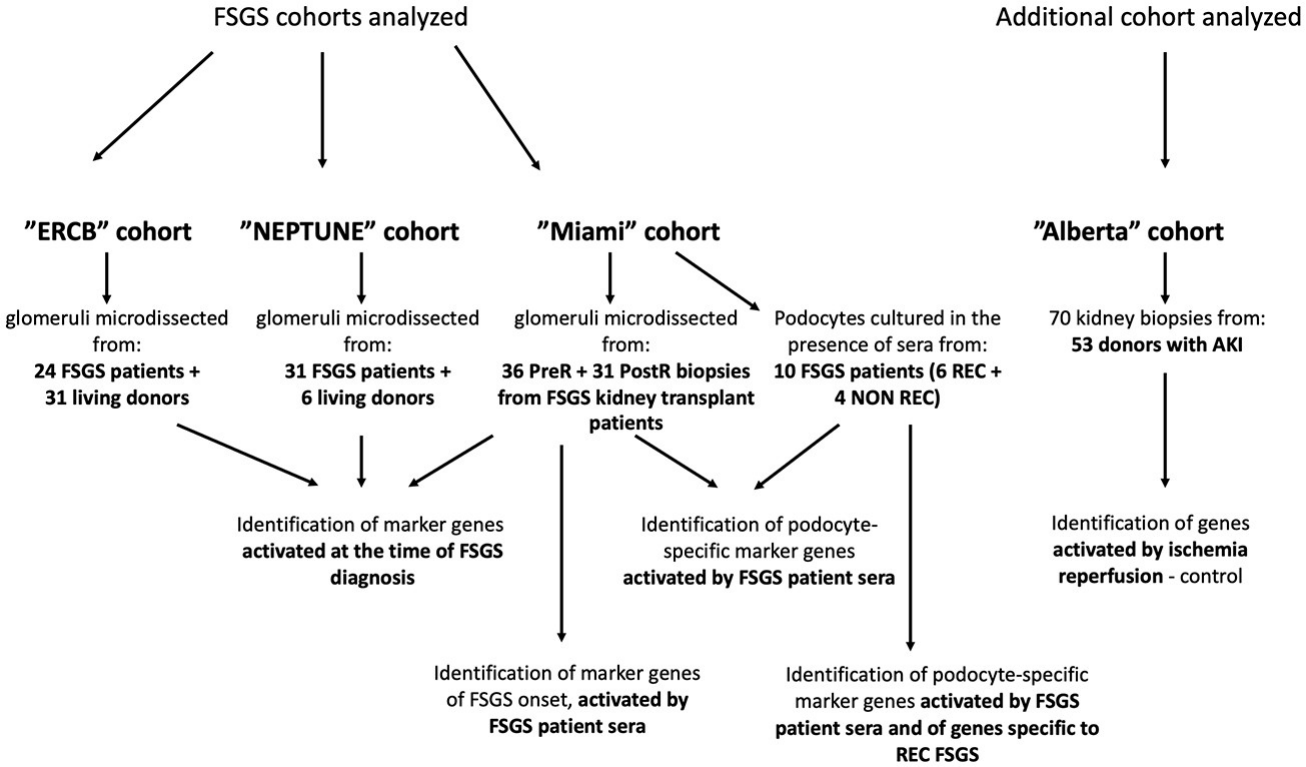
Strategic workflow. Microarray data from four different cohorts (ERCB, NEPTUNE, Miami, Alberta) were analyzed and compared to identify marker genes that are activated in glomeruli or podocytes during early FSGS pathogenesis, at the time of FSGS diagnosis and to identify markers specific to the recurrence of FSGS (Fig 1).

We implemented an unbiased approach in which we compared the microarray-derived molecular signatures of glomeruli obtained from the same transplanted kidney before transplant (PreR) as well as shortly after reperfusion (PostR). We also utilized patient sera from a previously published cohort to treat cultured human podocytes *in vitro* aimed at identifying clinically relevant druggable targets and patients at risk for REC after transplantation (Fig 1). Finally, microarray data from three additional cohorts (ERCB, NEPTUNE, Alberta) were analyzed and implemented in this study to identify genes activated in glomeruli or podocytes during early FSGS pathogenesis due to the presence of a circulating permeability factor(s) in patient sera, to identify genes activated at the time of diagnosis, and to identify markers specific to the REC (Fig 1). Baseline characteristics of the patients of the ERCB and NEPTUNE cohort are shown in S2 Table.

### Inflammatory signaling pathways are regulated in glomerular and tubulointerstitial PostR biopsies

**Fig 2:**
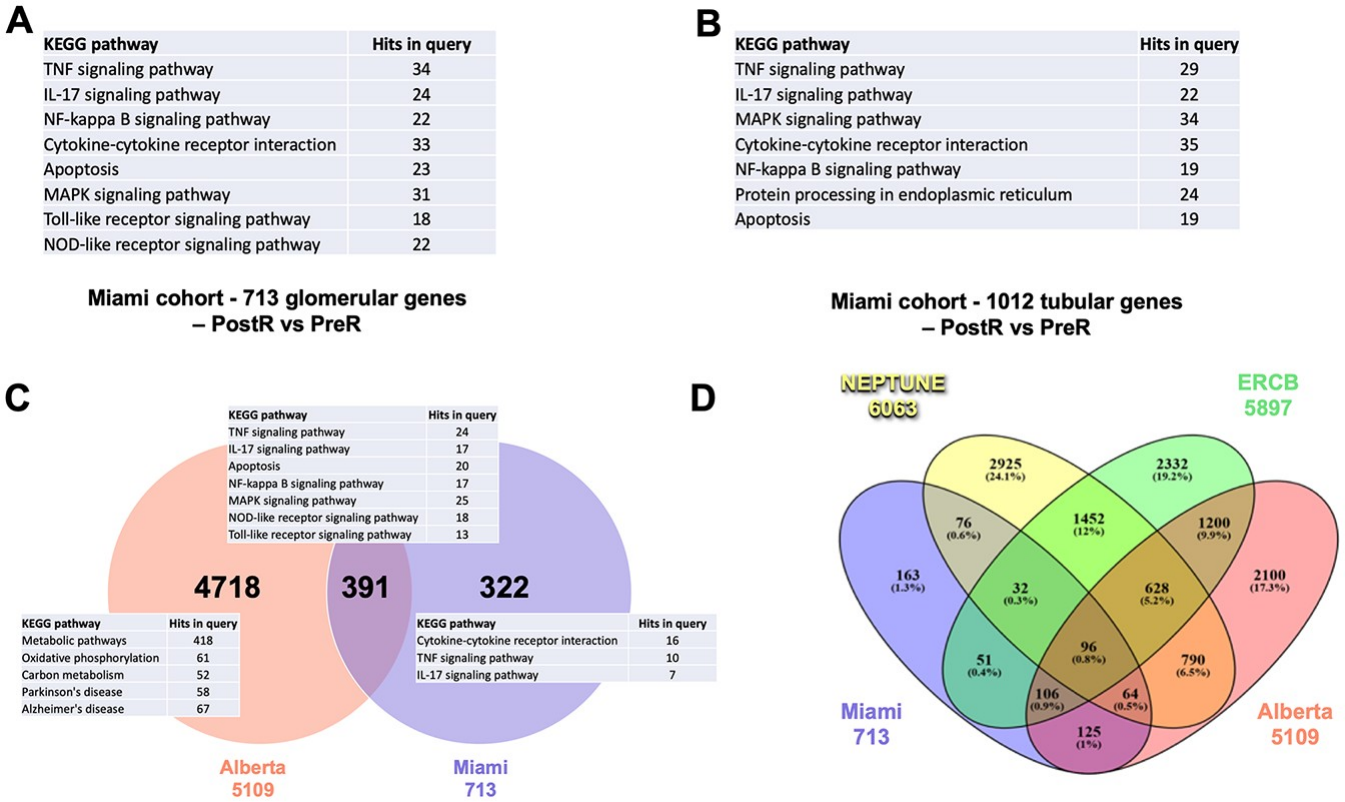
Identification of molecular pathways specifically activated in glomeruli of patients with NON REC FSGS. Microarray analysis, followed by gene enrichment KEGG pathway analysis of post (PostR) and pre- (PreR) reperfusion kidney biopsy samples obtained from patients with FSGS. **(A)** 713 genes are differentially regulated in the glomerular compartment of kidney biopsies obtained from patients with FSGS. KEGG pathway analysis identifies dysregulation of predominantly inflammatory as well as apoptotic pathways among the top 20 pathways. Selected pathways out of the top 20 are shown. **(B)** 1012 genes are differentially regulated in the tubulointerstitial compartment of kidney biopsies obtained from patients with FSGS. KEGG pathway analysis identifies dysregulation of predominantly inflammatory as well as apoptotic pathways among the top 20 pathways. Selected pathways out of the top 20 are shown. **(C, D)** Identification of differentially expressed genes and pathways specific to FSGS. **(C)** Glomerular gene expression changes identified in PostR vs PreR biopsies compared to genes differentially expressed in PostR biopsies from 70 kidneys from 53 deceased donors published in the “Alberta” study (GEO accession: GSE37838) are shown. Venn diagram analysis indicating that out of the 713 glomerular genes regulated in PostR vs PreR glomerular biopsies of FSGS patients, 322 genes are uniquely regulated in PostR biopsies of FSGS patients whereas 391 genes are also regulated in kidney biopsies of the Alberta patients. Inflammatory pathways are primarily activated in glomerular biopsies of the Miami cohort, metabolic pathways are particularly activated in the Alberta cohort of patients evaluated for acute kidney injury **(C)**. Venn Diagram analysis demonstrating differentially expressed genes specific to FSGS **(D)**.

A total of 36 PreR and 31 PostR kidney biopsies from 49 patients were available for microarray analysis (Miami cohort). Samples from patients with REC (6 PreR and 5 PostR) and NON REC (30 PreR and 26 PostR) were separated for the analysis. Unfortunately, due to the limited availability of samples from patients with REC, we were not able to detect statistically significant gene expression changes when analyzing the 5 PostR and 6 PreR biopsy samples from this group. However, we found differential expression of 713 genes in the glomerular (Fig 2A, S1 Table) and of 1012 genes in the tubulointerstitial compartment (Fig 2B, S1 Table) when analyzing PostR compared to PreR biopsies obtained from patients with NON REC of FSGS (FDR ≤ 0.05, p<0.05). Gene enrichment KEGG pathway analysis demonstrated predominant activation of inflammatory pathways among the top 20 regulated pathways in the glomerular and tubulointerstitial compartment (Fig 2A and 2B) indicating an important role for inflammation in early FSGS pathogenesis. Additionally, apoptotic pathways were also specifically activated in glomerular and tubular biopsies.

### Identification of glomerular genes and pathways specifically activated by FSGS patient sera

Inflammatory pathways play an important role in the development of ischemia reperfusion injury of the kidney as a result of prolonged oxygen deprivation of the kidney during transplantation. To identify pathways that are specifically activated due to the contact of the kidney with FSGS patient sera rather than being the consequence of the ischemia/reperfusion kidney injury or due to early activation of non-self-recognition pathways and cell-death, glomerular genes differentially regulated in PostR biopsies of the Miami cohort were compared to genes differentially expressed in PostR kidney biopsies from 70 kidneys from 53 deceased donors published in the Alberta study (Alberta cohort) (Fig 2C). We found that 391 of the 713 genes differentially regulated in PostR biopsies from patients with FSGS were also differentially regulated in PostR biopsies of the Alberta cohort (Fig 2C, S1 Table). Gene enrichment KEGG pathway analysis revealed that significantly regulated pathways include TNF, IL-17, NF-kappa B, MAPK, NOD-like receptor, TLR4 and apoptosis signaling pathways indicating that dysregulation of some genes within these pathways may be the result of kidney ischemia and reperfusion injury. However, when genes differentially regulated in the Alberta cohort were analyzed independently, metabolic pathways were predominantly, while inflammatory pathways were not significantly regulated (Fig 2C). More importantly, we identified 322 genes that were uniquely regulated in PostR biopsies of the Miami cohort of patients with FSGS (Fig 2C, S1 Table). Gene enrichment KEGG pathway analysis indicated that, even after removal of genes that might be activated due to ischemia/reperfusion injury or due to early activation of non-self-recognition pathways and cell-death, inflammatory signaling pathways remained the predominantly activated pathways in normal kidneys after exposure to FSGS patient sera (Fig 2C), confirming a role for these pathways in the early pathogenesis of primary FSGS. Further analysis of the individual genes is warranted to assess their specific contribution to early disease pathogenesis.

### Identification of markers and pathways activated by FSGS sera that remain activated after proteinuria becomes clinically evident

**Fig 3:**
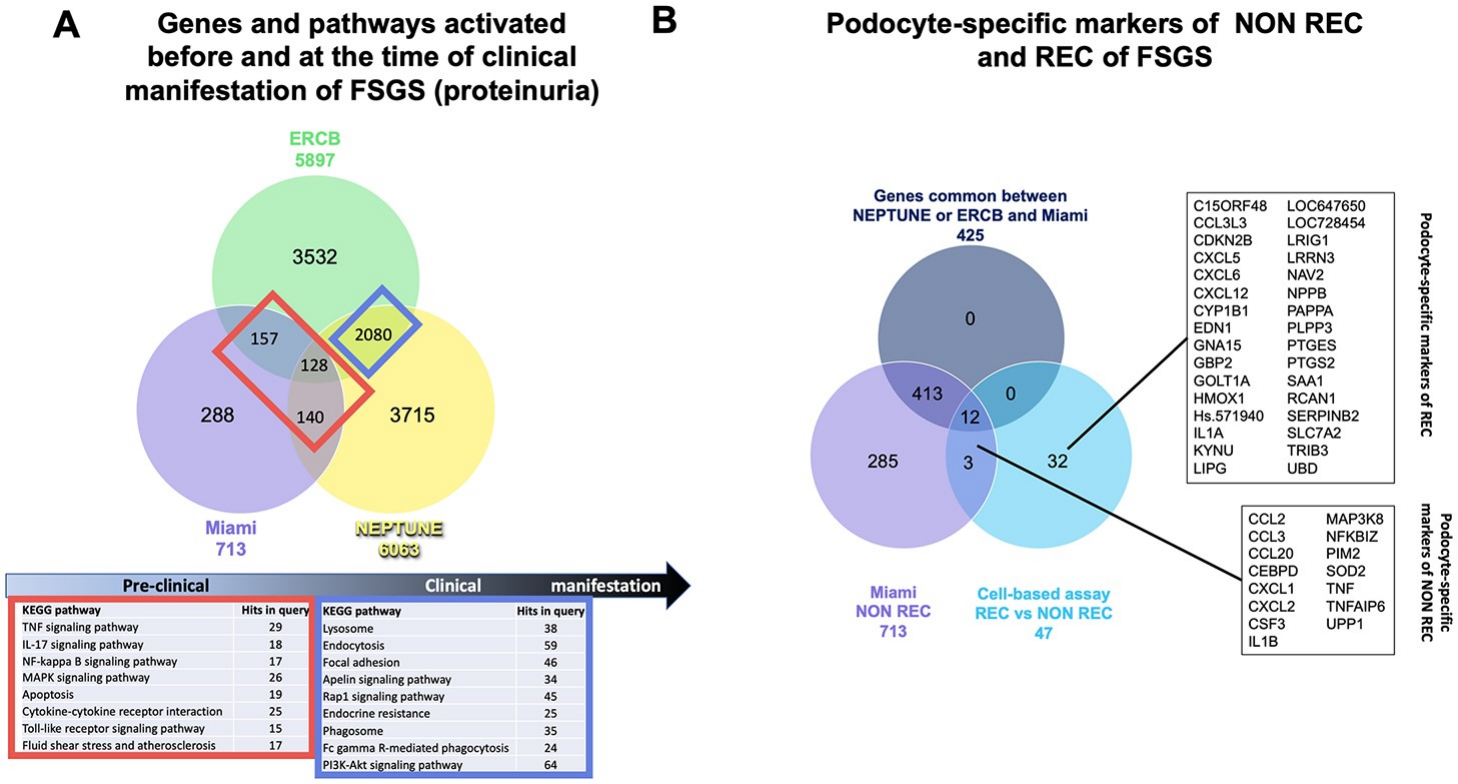
Identification of glomerular markers of primary FSGS and of podocyte-specific markers of non-recurrent and recurrent FSGS. (A) Venn diagram analysis of the gene expression profiles obtained from glomerular biopsies of patients with FSGS from the NEPTUNE cohort and of the ERCB cohort demonstrating that 128 genes are commonly regulated in all three cohorts, while 157 genes are commonly regulated between the Miami and the ERCB and 140 genes between the Miami and the NEPTUNE cohort. 2080 genes are commonly regulated between the ERCB and NEPTUNE cohorts. KEGG pathway analysis indicates the activation of inflammatory pathways in early FSGS progression and of phagocytotic pathways in later progression. (B) Microarray analysis identifies podocyte-specific genes regulated in podocytes cultured in the presence of sera from healthy subjects and from patients with REC or NON REC FSGS. Validation of the cell-based assay was obtained through comparison to the gene expression profiles obtained by analysis of glomerular biopsies obtained from the Miami, NEPTUNE and ERCB cohorts. Microarray analysis identifies 15 podocyte specific genes of NON REC FSGS and 33 genes were specifically regulated in human podocytes contacted with the sera from patients with REC compared to NON REC. Abbreviations of gene symbols are listed in Table 3.

To determine which of the genes/pathways modulated by exposure to patient sera are also activated in glomeruli of patients with active incident FSGS, gene expression profiles obtained from glomeruli of PostR biopsies of this study were compared to those of two other studies in which transcriptional profiling of patients with primary FSGS was performed, the NEPTUNE and ERCB cohorts (Figs 2D and 3A). Gene expression profiles of glomerular biopsies from 31 patients with FSGS and six living donor controls were available from the NEPTUNE study and from 24 patients with FSGS and 31 living donors of the ERCB study. We found 288 genes uniquely regulated in PostR biopsies of the Miami cohort suggesting that dysregulation of these genes occurs after the exposure to FSGS sera. Gene enrichment analysis did not indicate the activation of specific pathways. Additionally, we found 425 genes commonly dysregulated between glomeruli of the NEPTUNE and/or ERCB (Figs 2D and 3A) and our cohort. Of these, 140 genes were common between the Miami and the NEPTUNE cohort, 157 genes were common between the Miami and the ERCB cohort, and 128 genes were common between all three cohorts, suggesting an important role for these genes in the pathogenesis of FSGS. As expected, the overlap between differentially expressed genes in the NEPTUNE and the ERCB cohort (n = 2,080) was higher, suggesting that a larger number of glomerular genes is activated at time of clinical manifestation of the disease, and that the activation of these genes may be either the cause or the consequence of the disease. Interestingly, gene enrichment analysis indicated that pathways activated in early FSGS pathogenesis and before the clinical manifestation of the disease include predominantly inflammatory pathways while pathways that are activated at the time of clinical manifestation of FSGS include lysosomal, endocytic and phagosomal pathways, GTPase signaling and focal adhesion pathways (Figs 2D and 3A). The possibility that some of these genes are also activated in other glomerular diseases such as minimal change disease, membranous nephropathy or others remains to be established.

**Table 3:** List of gene symbols.

Symbol	Gene ID	Name
**C15ORF48**	84419	chromosome 15 open reading frame 48
**CCL2**	6347	chemokine (C-C motif) ligand 2
**CCL20**	6364	chemokine (C-C motif) ligand 20
**CCL3**	6348	chemokine (C-C motif) ligand 3
**CCL3L3**	414062	chemokine (C-C motif) ligand 3-like 3
**CDKN2B**	1030	cyclin-dependent kinase inhibitor 2B (p15, inhibits CDK4)
**CEBPD**	1052	CCAAT/enhancer binding protein (C/EBP), delta
**CSF3**	1440	colony stimulating factor 3 (granulocyte)
**CXCL1**	2919	chemokine (C-X-C motif) ligand 1 (melanoma growth stimulating activity, alpha)
**CXCL12**	6387	chemokine (C-X-C motif) ligand 12
**CXCL2**	2920	chemokine (C-X-C motif) ligand 2
**CXCL5**	6374	chemokine (C-X-C motif) ligand 5
**CXCL6**	6372	chemokine (C-X-C motif) ligand 6
**CYP1B1**	1545	cytochrome P450, family 1, subfamily B, polypeptide 1
**EDN1**	1906	endothelin 1
**GBP2**	2634	guanylate binding protein 2, interferon-inducible
**GNA15**	2769	guanine nucleotide binding protein (G protein), alpha 15 (Gq class)
**GOLT1A**	127845	golgi transport 1A
**HMOX1**	3162	heme oxygenase 1
**Hs.571940**		not available
**IL1A**	3552	interleukin 1, alpha
**IL1B**	3553	interleukin 1, beta
**KYNU**	8942	kynureninase
**LIPG**	9388	lipase, endothelial
**LOC647650**	647650	hypothetical protein LOC647650
**LOC728454**		hypothetical protein LOC28454
**LRIG1**	26018	leucine-rich repeats and immunoglobulin-like domains 1
**LRRN3**	54674	leucine rich repeat neuronal 3
**MAP3K8**	1326	mitogen-activated protein kinase kinase kinase 8
**NAV2**	89797	neuron navigator 2
**NFKBIZ**	64332	nuclear factor of kappa light polypeptide gene enhancer in B-cells inhibitor, zeta
**NPPB**	4879	natriuretic peptide B
**PAPPA**	5069	pregnancy-associated plasma protein A, pappalysin 1
**PIM2**	11040	Pim-2 proto-oncogene, serine/threonine kinase
**PLPP3**	8613	phospholipid phosphatase 3
**PTGES**	9536	prostaglandin E synthase
**PTGS2**	5743	prostaglandin-endoperoxide synthase 2 (prostaglandin G/H synthase and cyclooxygenase)
**RCAN1**	1827	regulator of calcineurin 1
**SAA1**	6288	serum amyloid A1
**SLC7A2**	6542	solute carrier family 7 (cationic amino acid transporter, y+ system), member 2
**SERPINB2**	5055	serpin peptidase inhibitor, clade B (ovalbumin), member 2
**SOD2**	6648	superoxide dismutase 2, mitochondrial
**TNF**	7124	tumor necrosis factor
**TNFAIP6**	7130	tumor necrosis factor, alpha-induced protein 6
**TRIB3**	57761	tribbles pseudokinase 3
**UBD**	10537	ubiquitin D
**UPP1**	7378	uridine phosphorylase 1

### Identification of podocyte-specific markers of primary FSGS

To identify podocyte-specific markers contributing to the pathogenesis of primary FSGS, microarray analysis of normal human podocytes contacted with the sera from patients with REC (n = 6) or NON REC (n = 4) (Fig 3B) FSGS was performed. The sera used were previously described in detail[5]. We identified 47 genes differentially expressed in podocytes treated with sera from patients with REC when compared to NON REC FSGS (Fig 3B, S1 Table). While pathway analysis did not reveal significant activation of a particular pathway due to the small number of genes, it is evident that many of these genes code for proteins with cytokine or chemokine activity (CCL2, CCL3, CCL3L3, CCL20, CXCL1, CXCL2, CXCL5, CXCL6, CXCL12, CSF3, EDN1, IL1A, IL1B, TNF). Venn diagram analysis was performed to determine if these genes are activated only in REC FSGS by implementing the set of genes that we found commonly activated in glomerular biopsies of the Miami, NEPTUNE and ERCB cohort. We identified 15 genes differentially expressed in both, PostR biopsies and in sera treated podocytes (Fig 3B) indicating that this cell-based assay could represent a valuable and non-invasive tool allowing for the identification of podocyte-specific gene expression changes that occur early during the pathogenesis of the disease and before the manifestation of clinical FSGS. While 3 genes, namely CCL3, CSF3, and TNFAIP6 were only activated in the cell-based assay and when PreR and PostR biopsies of the Miami cohort were analyzed, 12 genes, CCL2, CCL20, CEBPD, CXCL1, CXCL2, IL1B, MAP3K8, NFKBIZ, PIM2, SOD, TNF and UPP were also found activated in glomerular biopsies of the NEPTUNE and the ERCB cohorts. These data suggest that CCL3, CSF3 and TNFAIP6 may be podocyte specific markers which are activated rapidly after the exposure to a circulating factor(s) present in the sera of patients with FSGS but that do not remain activated at the stage of clinical manifestation of the disease, while the other 12 genes remain activated.

### Identification of genes that may be utilized to stratify patients at risk for REC

Finally, the same cell-based assay also allowed for the identification of 32 genes specifically regulated in podocytes cultured in the presence of sera from patients with REC (Fig 3B). Podocyte-specific genes that may be utilized to stratify patients at risk for REC included, amongst others, genes with a function in G-protein-coupled receptor binding (SAA1, GNA15, EDN1, CXCL12), genes with cytokine or chemokine activity (IL1A, CXCL5, CXCL6, CCL3L3), genes that are activated in response to cytokines (PTGES2, GBP2, UBD, KYNU, PTGS2, EDN1, CXCL2), genes involved in NFAT signaling (GNA15 and RCAN1) and genes in lipid metabolism, GOLT1A. However, for four genes, namely Hs. 571940, LOC647650, LOC728454 and IL1A, no expression data were available from the Miami cohort, thus, if these genes might play a role in the REC of FSGS needs to be further investigated. Taken together, these results suggest that these 32 genes may contribute to REC of FSGS and may help to stratify patients at risk for REC. However, a validation of these results in larger cohorts is needed.

### The expression of genes regulated in PostR glomeruli partially correlates with TNF expression and/or with clinical outcome

We previously reported that glomerular TNF expression in patients with primary FSGS correlates with loss of GFR[19], and our extensive microarray analysis of several cohorts with patients affected by FSGS in this study confirmed an important role of the TNF signaling pathway as a pathway that is activated prior to the clinical manifestation of the disease. We next performed correlation analysis between the 15 podocyte-specific genes (common between glomerular biopsies and the cell-based assay) identified differentially expressed in PostR biopsies and glomerular TNF expression (Table 4) as well as with clinical parameters (Table 5) of podocyte injury such as FPE, UPCR and loss of eGFR at 12 months. We found that the expression of 11 of the 15 genes commonly regulated between PostR biopsies of patients with primary FSGS and in the cell-based assay correlated with glomerular TNF expression, further confirming an important role for TNF and of these genes in disease pathogenesis (Tables 4 and 5). As expected, the expression of more than half of these genes (10/15) also correlated with clinical and morphological parameters, mostly with aberrant foot process width but also with UPCR and GFR in patients with primary FSGS (Tables 4 and 5).

**Table 4:** Differential gene expression in PostR biopsies of patients with FSGS partially correlates with local TNF expression and with clinical parameters.

Gene Symbol	Gene ID	TNF	DFPE	DFPW	UPCR	eGFR
**TNF**	7124					
**CCL2**	6347					
**CCL20**	6364					
**CCL3**	6348					
**CEBPD**	1052					
**CXCL1**	2919					
**CXCL2**	2920					
**IL1B**	3553					
**MAP3K8**	1326					
**NFKBIZ**	64332					
**PIM2**	11040					
**SOD2**	6648					
**CSF3**	1440					

Red boxes—positive correlation, green boxes—negative correlation, grey boxes—no significant correlation.

DFPE = difference in foot process effacement measured in PreR and PostR biopsies; DFPW = difference in foot process width measured in PreR and PostR biopsies; UPCR = urinary protein-to-creatinine ratio, eGFR = estimated glomerular filtration rate. TNF—tumor necrosis factor, CCL2—C-C Motif Chemokine Ligand 2, CCL20—C-C Motif Chemokine Ligand 20, CCL3—C-C Motif Chemokine Ligand 3, CEBPD—CCAAT/Enhancer Binding Protein Delta, CXCL1—chemokine (C-X-C motif) ligand 1 (melanoma growth stimulating activity, alpha), CXCL2—chemokine (C-X-C motif) ligand 2, IL1B—interleukin 1, beta, MAP3K8—Mitogen-Activated Protein Kinase Kinase Kinase 8, NFKBIZ—NFKB Inhibitor Zeta, PIM2—Pim-2 Proto-Oncogene, Serine/Threonine Kinase, SOD2—Superoxide Dismutase 2, CSF3—Colony Stimulating Factor 3.

**Table 5:** Differential gene expression in PostR biopsies of patients with FSGS partially correlates with clinical parameters.

Gene Symbol	Gene ID	TNF		FP		UPCR		eGFR	
		P	R2	P	R2	P	R2	P	R2
**TNF**	7124					0.0136*	0.237		
**CCL2**	6347	0.0005	0.4058	0.0373#	0.2194	0.0042**	0.3055		
				0.0076##	0.3343	0.0122***	0.2436		
						0.0414****	0.1758		
**CCL20**	6364	0.0179	0.2122	0.0060##	0.3495				
**CCL3**	6348	0.0035	0.3043						
**CEBPD**	1052	0.0013	0.3568	0.0057##	0.3537	0.0398**	0.1711		
**CXCL1**	2919	0.0014	0.351						
**CXCL2**	2920	<0.0001	0.6067					0.0255^	0.4551
**IL1B**	3553	0.0288	0.1839	0.0351##	0.2239	0.0306**	0.1874		
						0.0242***	0.2021		
**MAP3K8**	1326	0.0013	0.6317	0.0050##	0.362				
**NFKBIZ**	64332	<0.0001	0.6317	0.0330##	0.2287	0.0288*	0.1993		
						0.0253**	0.1993		
						0.0491***	0.158		
**PIM2**	11040	0.0141	0.226	0.0119##	0.3028				
**SOD2**	6648	0.0018	0.3383	0.0289#	0.2384				
				0.0026##	0.4038				
**CSF3**	1440			0.0051##	0.3607				
				0.0064###	0.4468				

Red boxes—positive correlation, green boxes—negative correlation, grey boxes—no significant correlation. FP = foot process; UPCR = highest protein-to-creatinine ratio between two time points, eGFR = glomerular filtration rate.

*highest UPCR measured between days 3–30,

** highest UPCR measured between months 1–12,

*** highest UPCR measured between months 3–12,

****UPCR at 3 months,

^difference in eGFR between months 1–3,

^#^difference in foot process effacement (DFPE) between PreR and PostR biopsies measured by electron microscopy,

^##^difference in foot process width (DFPW) between PreR and PostR biopsies measured by electron microscopy,

^###^foot process width in PostR biopsy.

Red boxes—positive correlation, green boxes—negative correlation, grey boxes—no significant correlation. TNF—tumor necrosis factor, CCL2—C-C Motif Chemokine Ligand 2, CCL20—C-C Motif Chemokine Ligand 20, CCL3—C-C Motif Chemokine Ligand 3, CEBPD—CCAAT/Enhancer Binding Protein Delta, CXCL1—chemokine (C-X-C motif) ligand 1 (melanoma growth stimulating activity, alpha), CXCL2—chemokine (C-X-C motif) ligand 2, IL1B—interleukin 1, beta, MAP3K8—Mitogen-Activated Protein Kinase Kinase Kinase 8, NFKBIZ—NFKB Inhibitor Zeta, PIM2—Pim-2 Proto-Oncogene, Serine/Threonine Kinase, SOD2—Superoxide Dismutase 2, CSF3—Colony Stimulating Factor 3.

## Discussion

This study aimed at identifying glomerular and podocyte-specific molecular markers that are activated by the exposure of the kidney to the sera of patients with primary FSGS. While several circulating factors were proposed to contribute to the pathogenesis of primary FSGS and REC of FSGS [12–15, 28, 29], it has become clear that primary FSGS is a heterogenous condition which is likely caused by multiple rather than a single circulating factor[14, 15].

We therefore implemented an unbiased approach in which we compared the microarray-derived molecular signatures of glomeruli obtained from the same transplanted kidney before transplant (PreR) as well as shortly after reperfusion (PostR).

One advantage of this approach is that it allows for the identification of molecular pathways that are activated by the sera of kidney transplant recipients with FSGS immediately after kidney transplantation, irrespectively of the presence and the nature of circulating permeability factors. This seems especially useful as it was previously suggested that multiple circulating factors might contribute additively or synergistically to the pathogenesis of primary FSGS, as described for TNF and suPAR in experimental studies using mouse podocytes[20].

Another advantage of this approach is that the comparison with additional cohorts that allowed us to discriminate FSGS sera induced pathways from ischemia-reperfusion related pathways. Finally, because PreR and PostR biopsies are obtained from the same kidney, gene expression variations due to differences in the genetic background of the patients can be eliminated, while such differences may contribute to some of the changes in gene expression when kidney biopsies from patients with FSGS are compared to those obtained from healthy subjects as is often the case in other published studies. We also utilized sera from 6 patients with REC and 4 patients with NON REC [5] to treat cultured human podocytes *in vitro* with the goal to identify clinically relevant druggable targets and patients at risk for REC after transplantation (Fig 1).

A total of 36 PreR and 31 PostR biopsy samples was analyzed by microarray. We demonstrate that inflammatory pathways including TNF, IL-17, NFkappa B, MAPK, and apoptotic signaling pathways are activated in the glomerular and tubulointerstitial compartments within 1–2 hours after the exposure of the kidney to the sera of FSGS patients undergoing kidney transplantation (Fig 2A) suggesting that these pathways are activated by one or more circulating factors present in the sera of patients with FSGS. While our study does not identify which factor(s) in the sera of patients with FSGS contribute to the activation of these signaling pathways, it supports our previous observations which suggested an important role of local TNF pathway activation in podocyte injury. However, because the experimental approach chosen does not discriminate which factor(s) in the sera of patients with FSGS, it does also not allow to exclude a potential role for circulating TNF alone or as one of multiple circulating factors to the pathogenesis of primary FSGS as previously suggest by others[20]. Further in-depth analysis is warranted to address this question.

While previously published observations by others and us indicated a correlation between glomerular TNF pathway activation and eGFR in patients with primary FSGS[19], we were not able to detect a similar correlation in this post-transplant cohort. This may be explained by the relatively small sample size of 31 PostR kidney biopsies available for analysis and the short follow up of the patients for only up to 12 months, or it may simply suggest that TNF pathway activation is solely a susceptibility factor to post-transplant eGFR loss, and other contributing factors are involved. Additionally, results from the FONT study [17, 18] suggest that only a subset of patients with FSGS responds to TNF inhibition and Mariani et al. (2018) reported that only patients at high risk for FSGS progression (cluster 3 patients) are characterized by a transcriptional profile consistent with TNF activation that correlates with eGFR loss [21]. These observations might explain why we did not find a correlation between TNF and eGFR in our study as most of the patients in the present study were patients at low risk for REC and ultimately only four patients eventually had recurrence of proteinuria. However, we did find a positive correlation between TNF expression in PostR biopsies and the highest value of urinary albumin-to-creatinine ratio (UACR) measured in patients between days 3 and 30 after transplantation suggesting a possible role for TNF pathway activation in podocyte injury. Further comparison to gene expression data published in the Alberta study demonstrates that although some genes of the TNF pathway may be activated as a consequence of the reperfusion of the kidney, at least 16 genes of this pathway are specifically activated as a response to FSGS sera (Fig 2C). Additionally, the observation that the TNF signaling pathway is not significantly activated in the Alberta cohort, when analyzed by itself, further supports that this pathway is likely to be specifically activated by one or more circulating factors present in the sera of patients with FSGS rather than as a consequence of acute kidney injury, or of ischemia/reperfusion injury. We further confirmed that TNF signaling remains activated at the time of biopsy (established incident diagnosis of FSGS), as indicated by the analysis of glomerular biopsies from the NEPTUNE and ERCB studies (Fig 3A). Transcriptome comparison of the NEPTUNE, ERCB and Miami cohorts led to the identification of 425 genes that are activated shortly after the contact of the kidney with the sera of patients and that remain activated at the time of clinical manifestation of FSGS. Interestingly, we also identified a set of 2080 genes that was commonly activated between the NEPTUNE and ERCB cohorts but not in the Miami cohort (Fig 3A), indicating that these genes are likely not activated by the exposure of the kidney to patient sera. However, whether the activation of these genes is a cause of proteinuria or occurs as a consequence of established proteinuria remains to be established. To identify podocyte-specific markers activated by the exposure of the kidney to sera from patients with REC compared to NON REC, a cell-based assay was used in which human podocytes were cultured in the presence of sera from patients with or without recurrence of FSGS. Results obtained were compared to microarray results obtained when comparing glomerular PostR and PreR biopsies and glomerular biopsies of the ERCB and NEPTUNE cohorts. We were able to identify 15 podocyte-specific markers of FSGS that are activated by the presence of a circulating permeability factor(s) in the serum of patients with FSGS (Fig 3B). As expected, most of these genes were inflammatory markers including chemokine ligands and interacting proteins (*CCL2*, *CCL3*, *CCL20*, *CXCL1*, *CXCL2*, *TNFAIP6*), cytokines (*CSF3*, *IL1B*, *TNF*), proteins important in regulating the toll-like receptor (TLR) mediated innate immune response (*CEBPD*, *MAP3K8*, *NFKBIZ*) and other proteins (*PIM2*, *SOD2*, *UPP1*), supporting the concept that of one or more circulating factors present in the sera of patients with FSGS is responsible for the activation of inflammatory pathways. Furthermore, we also found dysregulation of 32 genes specifically dysregulated in podocytes that were incubated in the presence of the sera from patients with REC compared to NON REC, indicating that these genes may be activated by circulating factors that are only present in the sera of patients with REC of FSGS (Fig 3B). One of the major limitations of this study is, however, that it does not allow to discriminate between one or several circulating factors, or to determine the nature of this (these) circulating factor(s).

Many of the genes, especially inflammatory marker genes have been previously linked to and extensively studied in the pathogenesis of FSGS[23, 30, 31]. Therefore, their role in the pathogenesis of FSGS will not be discussed here in detail.

Finally, we demonstrated that 12 of the 15 genes that were commonly regulated between PostR biopsies of patients with FSGS and the cell-based assay correlated with glomerular TNF expression supporting our previous observation that TNF signaling may play an important role in the pathogenesis of FSGS. As expected, the expression of more than half of these genes (9/15) also correlated with clinical and morphological parameters, mostly with aberrant foot process width but also with UPCR and eGFR in patients with primary FSGS (Tables 4 and 5). However, due to the limited availability of PostR biopsy samples from patients with REC, the role of TNF signaling in REC of FSGS remains to be established in larger cohorts.

Our study has several limitations, i.e. only a limited number of patients were enrolled (39), most patients were adults (35.10 +/- 13.84 years) and a low rate of REC (4/39) after kidney transplantation was observed. In addition, the high number of adult patients, the relatively slow progression to ESRD (4.25 years ±4.18) and the high percentage of male patients (67%) enrolled in the study may have contributed to the low rate of REC. Furthermore, limited sample availability did not allow for validation of microarray-based results and the overall limited number of patients enrolled with available post-reperfusion biopsies prevented us from performing further in-depth analysis of subsets of patients, the microarray analysis of podocytes contacted with the sera from patients with REC and NON REC FSGS in the cell-based assay allowed for the identification of genes that are activated by the sera of patients with FSGS and also of genes that are specifically activated REC sera are utilized. However, further in-depth analysis of these genes in order to determine their role in the REC of FSGS is warranted. Nevertheless, although the number of patients enrolled in this cohort is limited, to our knowledge, it represents the largest collection of PreR and PostR kidney biopsies from transplanted patients affected by FSGS. Therefore, our study offers the largest microarray data collection of PreR and PostR kidney biopsies from patients with FSGS available to date. Furthermore, gene expression profiling in combination with gene enrichment pathway analysis enabled us to identify several podocyte-specific markers and pathways that may play a role in the early pathogenesis of FSGS as they are activated by circulating factors present in the sera of kidney transplant recipients with FSGS immediately after kidney transplantation. While some of these genes were extensively studied by us and others with regard to their role in the pathogenesis or progression of FSGS, little is known about many other genes. Therefore, this study lays the ground to study the contribution of these genes to the pathogenesis of FSGS in more detail and opens the possibly for the identification of new drug targets for the treatments of patients with FSGS.

Lastly, the correlation of many of these genes with glomerular TNF expression in patients with FSGS offers the first clinical validation of our experimental findings describing an important role of the TNF pathway in the pathogenesis of FSGS. The correlation of some of these genes with distinct clinical and morphological parameters of FSGS may help differentiate pathways relevant to podocyte injury and proteinuria from pathways relevant to loss of GFR.

## Supporting information

S1 Fig Schematic representation of wedge biopsy procurement and processing of donor kidneys. (A) Schematic representation of wedge biopsy procurement of Pre- and Post reperfusion kidney biopsies. (B) Inter-operatively obtained wedge biopsies of donor kidneys were cut in four pieces. Pieces were submerged in 2.5% glutaraldehyde fixative for Electron Microscopy (EM) analysis, 10% buffered formalin solution for histological analysis by light microscopy (LM), in Michel’s transport medium followed by embedding in optimum cutting temperature (*O*.*C*.*T*.) formulation for immunofluorescence (IF) and in RNA later for microarray analysis (MA).
(TIFF)

S1 Table Overlap in gene expression between different cohorts of the study. (XLSX)

S2 Table Baseline characteristics of the patients of the ERCB and NEPTUNE cohorts. (DOCX)

S1 File (A) Supporting Methods. (B) Members and contributing centers of the Nephrotic Syndrome Study Network.
(DOCX)
