# Supplementary figures and images for: Identification of glomerular and podocyte-specific genes and pathways activated by sera of patients with focal segmental glomerulosclerosis
Source: PLoS One. 2019 Oct 3;14(10):e0222948. doi: 10.1371/journal.pone.0222948 (PMC6776339; doi:10.1371/journal.pone.0222948)

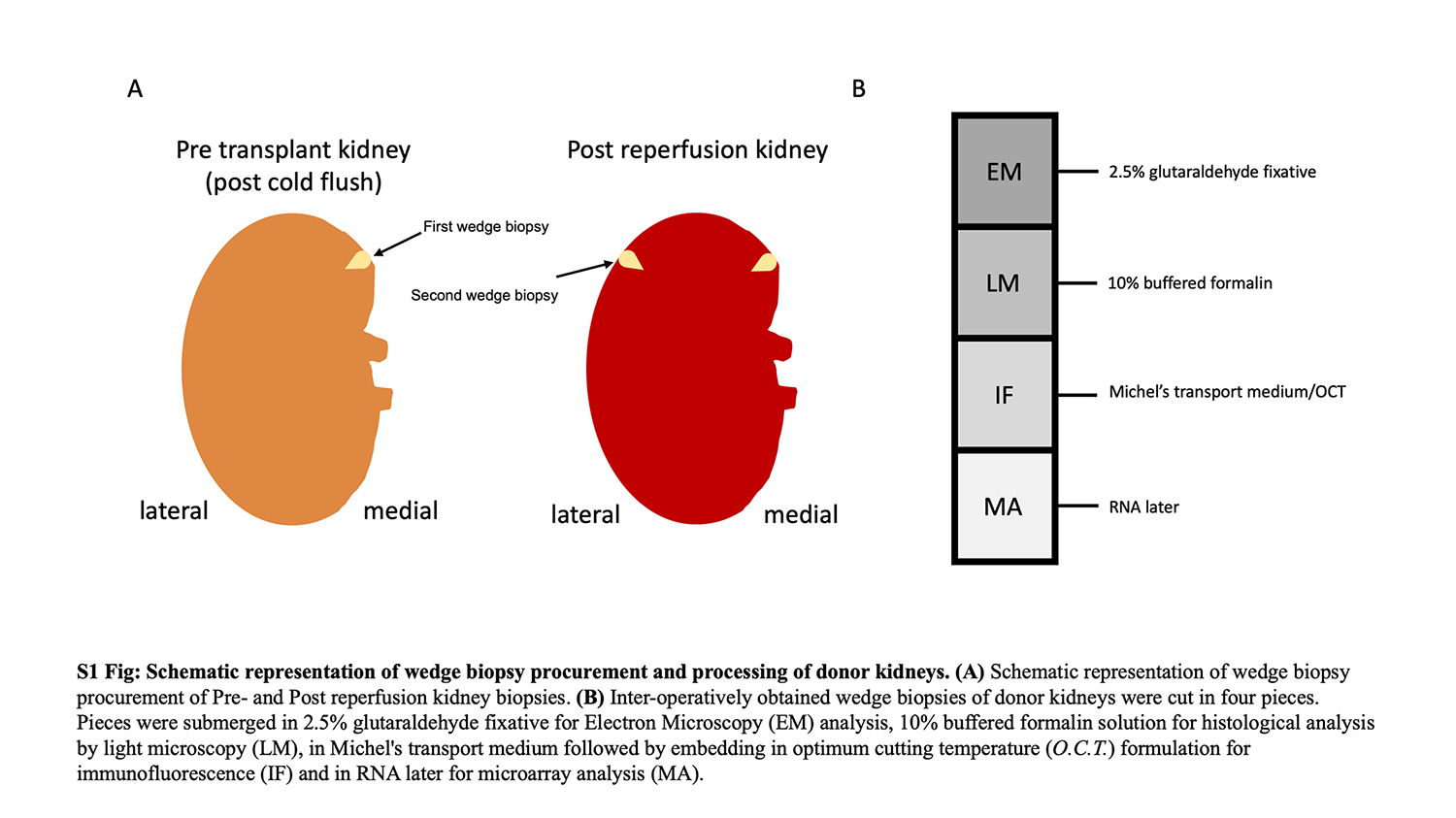

Supplement: S1 Fig — (A) Schematic representation of wedge biopsy procurement of Pre- and Post reperfusion kidney biopsies. (B) Inter-operatively obtained wedge biopsies of donor kidneys were cut in four pieces. Pieces were submerged in 2.5% glutaraldehyde fixative for Electron Microscopy (EM) analysis, 10% buffered formalin solution for histological analysis by light microscopy (LM), in Michel’s transport medium followed by embedding in optimum cutting temperature (O.C.T.) formulation for immunofluorescence (IF) and in RNA later for microarray analysis (MA). (TIFF) [file pone.0222948.s001.tiff]
