# Supplementary material for: Identification of glomerular and podocyte-specific genes and pathways activated by sera of patients with focal segmental glomerulosclerosis
Source: PLoS One. 2019 Oct 3;14(10):e0222948. doi: 10.1371/journal.pone.0222948 (PMC6776339; doi:10.1371/journal.pone.0222948)
Supplement: S2 Table — (DOCX) [file pone.0222948.s003.docx]

## **S2 Table. Baseline characteristics of the patients of the ERCB and NEPTUNE cohorts.**

|  | ERCB | Neptune |
| --- | --- | --- |
| Age | 45.52(±15.16) | 43.27(±16.37) |
| Sex | 12M/12F | 26M/5F |
| Race (W/B/O) | 24/0/0 | 19/8/4 |
| Weight (kg) | 77.36(±10.07) | 98.83 (±25.81) |
| Proteinuria (mg/mg) | 4.73(±3.23) | 2.0(±1.63) |
| eGFR, MDRD ml/min/1.73m^2^ | 74.78(±33.23) | 71.83(±30.19) |

eGFR = estimated glomerular filtration rate; MDRD = Modification of Diet in Renal Disease
