# Supplementary material for: Identification of glomerular and podocyte-specific genes and pathways activated by sera of patients with focal segmental glomerulosclerosis
Source: PLoS One. 2019 Oct 3;14(10):e0222948. doi: 10.1371/journal.pone.0222948 (PMC6776339; doi:10.1371/journal.pone.0222948)
Supplement: S1 File — (A) Supporting Methods. (B) Members and contributing centers of the Nephrotic Syndrome Study Network. (DOCX) [file pone.0222948.s004.docx]

# **S1 File**

1. **SUPPORTING METHODS**

**Exclusion criteria for patient enrollment and sample procuration.**

Exclusion criteria were seropositivity for human immunodeficiency virus (HIV), Hepatitis B or C virus, the presence or history of malignancies within the past 5 years, the presence of concomitant infections and/or severe diarrhea, vomiting, active upper gastro-intestinal tract malabsorption, the presence of an active peptic ulcer or any other unstable medical condition that could interfere with study objectives. Pregnant patients or patients with any form of substance abuse or psychiatric disorder and patients with a defined genetic cause of FSGS were also excluded. Genetic testing was not required as part of the inclusion criteria.

Morning random urine collections at day 3, 1 month, 3 months and 12 months were performed for the evaluation of protein and creatinine content and serum samples were collected at baseline, pre-transplantation and post-transplantation. The glomerular filtration rate (GFR) was determined using the Chronic Kidney Disease Epidemiology Collaboration (CKD-EPI) formula at day 3, 1 month, 3 months and 12 months. Samples were stored at -20 degrees and used for the experimental studies as described. Baseline demographic and clinical data were collected from the transplant chart by one investigator and are summarized in tables 1 and 2. An additional seven patients with biopsy proven FSGS that were enrolled in a prior study were also included in the present analysis^3^.

**Description of other cohorts used for comparative analyses in this study.**

Molecular profiles and gene expression data from 31 patients with a diagnosis of FSGS based on renal biopsies and from six living donors of the NEPTUNE cohort were implemented in our study. Eligibility criteria for enrollment of patients in the NEPTUNE study are detailed at <https://repository.niddk.nih.gov/studies/neptune/?query=None>. Baseline characteristics of the patients are summarized in S1 Table.

Similarly, molecular profiles and gene expression data from 24 patients with biopsy proven FSGS and 31 living donors of the European Renal Biopsy cDNA Bank (ERCB) cohort were included. Baseline characteristics of the patients are summarized in S1 Table.

Publicly available microarray data of the “Alberta” study, in which PostR implantation biopsies taken during reperfusion in 70 kidneys from 53 deceased donors due to acute kidney injury (AKI) was analyzed^39^ (GEO accession: GSE37838), were also implemented.

**EM examination of changes in FPs**

The specimens for EM were placed in glutaraldehyde solution at the time of biopsy. Images were taken at several magnifications using the digital image capture software (Philips CM-10). A total of four PreR and four PostR images per patient was selected by one investigator not involved in performing the actual study. Two blinded investigators independently traced and measured the length of the glomerular basement membrane (GBM) in selected images for a minimum of 200µm total per patient including at least 100µm of GBM for each, PreR and PostR biopsies. The length of the GBM was then analyzed using the image analysis software, ImageJ (NIH, Rockville, MD, USA). The total number of FPs was counted along the measured GBM and expressed as the number of FPs per micrometer GBM length for each image. The arithmetic mean of the foot process (FP) width (FPW) was calculated using the equation *FPW=π/4⋅∑GBM length/∑FPs* as previously published^24^. ∑GBM length represents the total GBM length, ∑FPs the total number of FPs measured in each picture and the correction factor of π/4 was used to correct for presumed random variation in the angle of sections relative to the long axis of the podocyte. A FP was defined as any connected epithelial segment abutting the basement membrane, separated from the cytoplasmic extensions of the adjacent FPs by lateral membranes^25^.

**Human podocyte culture**

Human podocytes were cultured at 33°C followed by differentiation at 37°C for 14 days as previously described^26^. Terminally differentiated podocytes were serum starved in 0.2% FBS for 24h and then exposed to 4% patient sera for and additional 24 hours as previously described^22,27^.

**Glomerular and tubulointerstitial mRNA preparation from kidney biopsies**

Kidney biopsy tissue for MA was manually microdissected into glomerular and tubulointerstitial compartments. RNA was extracted using the AllPrep RNA/DNA Micro 80284 Kit (Qiagen) following manufacturer’s instructions. Complementary DNA (cDNA) was prepared from approximately 10-15ng total RNA using the NuGen RNA cDNA Pico SL WTA system V2. 2.5 µg of cDNA was biotinylated using the NuGEN Encore Biotin Module (Encore Biotin Module Manual, P/N M01111 v6).

1. **MEMBERS AND CONTRIBUTING CENTERS OF THE NEPHROTIC SYNDROME STUDY NETWORK**

*NEPTUNE Enrolling Centers*

*Case Western Reserve University, Cleveland, OH*: J Sedor^*^, K Dell**, M Schachere^#^, J Negrey

*Children’s Hospital, Los Angeles, CA*: K Lemley^*^, L Whitted^#^

*Children’s Mercy Hospital, Kansas City, MO*: T Srivastava^*^, C Haney^#^

*Cohen Children’s Hospital, New Hyde Park, NY:* C Sethna^*^, K Grammatikopoulos^#^, R Odusayana

*Columbia University, New York, NY:* G Appel^*^, M Toledo^#^

*Emory University, Atlanta, GA:* L Greenbaum^*^, C Wang**, B Lee^#^

*Harbor-University of California Los Angeles Medical Center:* S Adler^*^, C Nast^*‡^, J La Page^#^

*John H. Stroger Jr. Hospital of Cook County, Chicago, IL:* A Athavale^*^

*Johns Hopkins Medicine, Baltimore, MD:* A Neu^*^, S Boynton^#^

*Mayo Clinic, Rochester, MN:* F Fervenza^*^, M Hogan**, J Lieske^*^, V Chernitskiy^#^

*Montefiore Medical Center, Bronx, NY:* F Kaskel^*^, N Kumar^*^, P Flynn^#^

*NIDDK Intramural, Bethesda MD:* J Kopp^*^, E Castro-Rubio^#^, J Blake^#^

*New York University Medical Center, New York, NY:* H Trachtman^*^, O Zhdanova**, F Modersitzki^#^, S Vento^#^

*Stanford University, Stanford, CA:* R Lafayette^*^, K Mehta^#^

*Temple University, Philadelphia, PA:* C Gadegbeku^*^, D Johnstone**, S Quinn-Boyle

*University Health Network Toronto:* D Cattran^*^, M Hladunewich**, H Reich**, P Ling^#^, M Romano^#^

*University of Miami, Miami, FL:* A Fornoni^*^, L Barisoni^*^, C Bidot^#^

*University of Michigan, Ann Arbor, MI:* M Kretzler^*^, D Gipson*, A Williams^#^, R Pitter^#^

*University of North Carolina, Chapel Hill, NC:* V Derebail^*^, K Gibson^*^, S Grubbs^#^, A Froment^#^

*University of Pennsylvania, Philadelphia, PA:* L Holzman^*^, K Meyers**, K Kallem^#^, A Swensen^#^

*University of Texas Southwestern, Dallas, TX:* K Sambandam^*^, E Brown**, M Cruz^#^

*University of Washington, Seattle, WA:* A Jefferson^*^, S Hingorani**, K Tuttle**^§^, L Curtin^#^, S Dismuke^#^, A Cooper^#§^

*Wake Forest University, Winston-Salem, NC:* B Freedman^*^, JJ Lin**, M Spainhour^#^, S Gray^#^

*Data Analysis and Coordinating Center*: M Kretzler, L Barisoni, C Gadegbeku, B Gillespie, D Gipson, L Holzman, L Mariani, M Sampson, P Song, J Troost, J Zee, E Herreshoff, S Li, C Lienczewski, T Mainieri, M Wladkowski, A Williams, D Zinsser

*National Institute of Diabetes and Digestive and Kidney Diseases (NIDDK) Program Office:* K Abbott, C Roy

*The National Center for Advancing Translational Sciences (NCATS) Program Office:* T Urv, PJ Brooks

*Principal Investigator; **Co-investigator^; #^Study Coordinator

^‡^Cedars-Sinai Medical Center, Los Angeles, CA

^§^Providence Medical Research Center, Spokane, WA
